# Do geography and ethnicity play a role in juvenile Spondyloarthritis? A multi-center binational retrospective study

**DOI:** 10.1186/s12969-020-00489-8

**Published:** 2021-01-06

**Authors:** Nassem Ghantous, Merav Heshin-Bekenstein, Kimberly Dequattro, Yaniv Lakovsky, Amir Moshe Hendel, Nadav Rappoport, Yonatan Butbul Aviel, Irit Tirosh, Liora Harel, Pamela F. Weiss, Lianne Gensler, John Mackenzie, Gil Amarilyo

**Affiliations:** 1 Sackler Faculty of Medicine, Tel Aviv University, Tel Aviv, Israel; 2 Dana Children’s Hospital, Tel Aviv Sourasky Medical Center, Tel Aviv, Israel; 3 School of Medicine, University of California, San Francisco, San Francisco, CA USA; 4 Schneider Children’s Medical Center of Israel, Petach Tikva, Israel; 5 Bakar Computational Health Sciences Institute, University of California, San Francisco, San Francisco, CA USA; 6 Rambam Medical Center, Haifa, Israel; 7 Sheba Medical Center, Tel Hashomer, Israel; 8 Children’s Hospital of Philadelphia, Perelman School of Medicine, University of Pennsylvania, Philadelphia, PA USA; 9 Pediatric Rheumatology Unit, Schneider Children’s Medical Center of Israel, 4920235 Petach Tikva, Israel

**Keywords:** Juvenile Spondyloarthritis, Enthesitis related arthritis, Juvenile Sacroiliitis, HLA B27

## Abstract

**Background:**

Observations among Israeli pediatric rheumatologists reveal that pediatric Juvenile Spondyloarthritis (JSpA) may present differently compared to patients from the United States (US). This study is aimed to compare the demographic and clinical variables of Israeli and US JSpA patients upon presentation.

**Methods:**

We performed a retrospective, cross-sectional, multicenter comparison of JSpA patients among 3 large Israeli pediatric rheumatology centers and a large US pediatric rheumatology center. Patients with diagnosis of Juvenile Ankylosing Spondylitis (JAS) and/or Enthesitis-related Arthritis (ERA) were included. The demographic, clinical and radiologic features were compared.

**Results:**

Overall 87 patients were included (39 Israeli, 48 US patients). Upon presentation, inflammatory back pain, sacroiliac joint tenderness and abnormal modified Schober test, were significantly more prevalent among Israeli patients (59% vs. 35.4, 48.7% vs. 16.7, and 41.2% vs. 21.5%, respectively, all *p* < 0.05), whereas peripheral arthritis and enthesitis were significantly more prevalent among US patients (43.6% vs. 91.7 and 7.7% vs. 39.6% in Israeli patients vs. US patients, p < 0.05). In addition, 96.7% of the Israeli patients versus 29.7% of the US patients demonstrated sacroiliitis on MRI (*p* < 0.001, *N* = 67). Less than one-third of the Israeli patients (32%) were HLA-B27 positive vs. 66.7% of US patients (*p* = 0.007).

**Conclusion:**

Israeli children with JSpA presented almost exclusively with axial disease compared to US patients who were more likely to present with peripheral symptoms. HLA B27 prevalence was significantly lower in the Israeli cohort compared to the US cohort. Further studies are needed to unravel the genetic and possibly environmental factors associated with these findings.

**Supplementary Information:**

The online version contains supplementary material available at 10.1186/s12969-020-00489-8.

## Background

Spondyloarthritis (SpA) represent a group of heterogeneous conditions characterized by arthritis, enthesitis and increased risk of axial disease, with similarities in the genetic susceptibility [1].

Although SpA usually begins in the third or fourth decade of life, 10–20% of patients experience symptoms during childhood, and the diagnosis of Juvenile Spondyloarthritis, (JSpA) accounts for 15–20% of the chronic arthritis diseases in children in North America and Europe [2]. The extra-articular manifestations of juvenile SpA may include: (1) Acute anterior uveitis (AAU), which is reported to occur in one quarter of children, similarly to adults [3], (2) Bowel inflammation, which is reported in approximately two thirds of children, similarly to adults [4, 5], (3) Psoriasis, in which prevalence is unclear among children with JSpA and is reported as 10–25% in adult SpA [6], and (4) Cardiovascular manifestations of conduction disturbances and aortic insufficiency, which are documented in adults but are rare among children [6, 7].

A single diagnostic system that is representative of the JSpA population is still lacking. In 2002, the International League against Rheumatism (ILAR) developed the currently used classification for Juvenile Idiopathic Arthritis (JIA). Enthesitis-related arthritis (ERA) which is a category of JIA, describes a disease which affects the joints and entheses and may involve the sacroiliac (SI) joints. The diagnosis of ERA requires the presence of arthritis and enthesitis, or arthritis or enthesitis with two or more minor criteria which may include sacroiliac symptoms [8, 9]. The main problem in this classification system is that patients with sole presentation of sacroiliitis, and less than 2 minor criteria, are excluded from the ERA per definition [1, 9]. Instead, these individuals are thus defined as undifferentiated JIA. Therefore, classification of JSpA according to the JIA classification system is inaccurate [10]. The term Juvenile Ankylosing Spondylitis (JAS) refers to the axial involvement of the disease, however, it is important to note that JAS definition is still unclear in the literature, and thus is not an official category of ILAR [10]. No specific criteria for sole axial involvement exist for children, and adoption of adult criteria is problematic because of the unique presentation of the disease in pediatric patients. Studies of North American and European JSpA patients have demonstrated that JSpA is strongly associated with the expression of class I MHC molecule HLA-B27, especially among those with axial involvement [11], and characterized by a high male to female ratio, and a peak age of onset at early adolescence [7]. In addition, those studies also showed low frequency of axial involvement (e.g. sacroiliitis) as a presenting symptom, unlike the adult type [2]. Children commonly present with peripheral arthritis and enthesitis affecting the lower extremities, whereas axial involvement has been reported in up to 30% of children within 15 months of diagnosis [11]. Clinical features associated with sacroiliitis in children are higher active joint and entheses counts at diagnosis, in addition to hip arthritis [12, 13]. Since clinical observations of Israeli pediatric rheumatologists demonstrated different demographic features and presenting symptoms of Israeli JSpA patients, our aim in this study is to characterize the demographic and clinical features of these patients and compare them to a cohort of patients from the United States (US).

## Methods

### Study site and participants

**Fig. 1:**
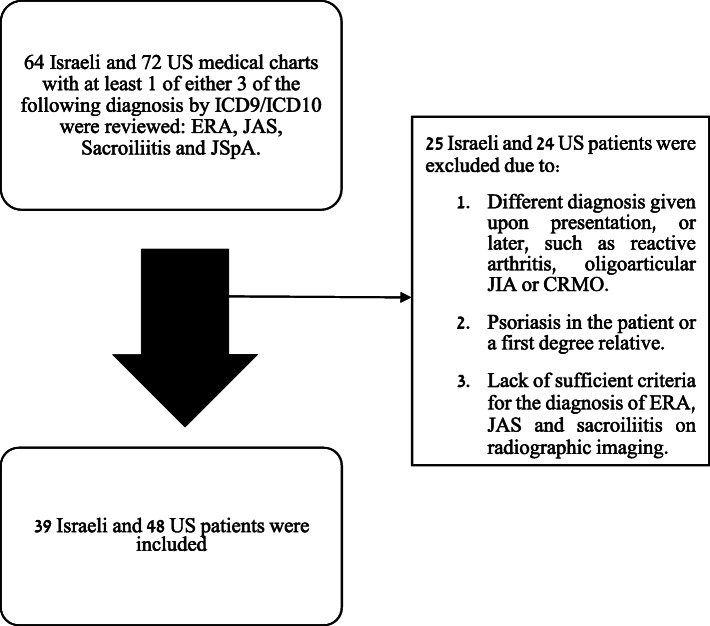
Flow diagram presenting the selection of study population. *ICD* International classification of disease, *ERA* Enthesitis-related arthritis, *JAS* Juvenile ankylosing spondylitis, *JSpA* Juvenile spondyloarthritis, *JIA* Juvenile idiopathic arthritis, CRMO Chronic recurrent multifocal osteomyelitis

We performed a retrospective cross-sectional study using data from three large pediatric rheumatology centers in Israel from 12/2004 to 09/2017. The study sites were Schneider Children’s Medical Center (Petah-Tekva, Israel), Sheba Medical Center (Tel Hashomer, Israel) and Rambam Health Care Campus (Haifa, Israel). As a comparison group, we used data from the large tertiary center, Benioff Children’s Hospital (University of California San Francisco, California (CA)) from 1/2013 to 12/2017. The protocol for this study was reviewed and approved by each of the committees for the protection of human subjects from the four institutions. All subjects met the following inclusion criteria: (1) Diagnosis of ERA according to ILAR criteria upon presentation (which is defined as meeting the ERA criteria within 3 months of initial presentation to the rheumatology clinic) OR diagnosis of unilateral or bilateral sacroiliitis, proved by MRI imaging, who did not meet the ILAR criteria for diagnosis of ERA, (2) Aged < 16 years at symptom onset. In order to ensure that all patients with axial involvement in all sites are included, we used all available codes for axial involvement. Exclusion criteria included: (1) Diagnosis with any other rheumatologic disease at the time of JSpA diagnosis or subsequently (e.g. reactive arthritis, chronic non-infectious osteomyelitis (CNO), Juvenile Psoriatic Arthritis), (2) Lack of sufficient criteria for diagnosis of ERA, JAS or sacroiliitis based on MRI imaging), and (3) Follow up documentation of less than 4 months of initial presentation. Patients from all sites were identified from the medical records using identical ICD 9 and 10 diagnostic codes (Supplementary Table 1) and chart review included all consecutive JIA patients who met the inclusion criteria. Each medical record was extensively reviewed by a pediatric rheumatologist (2 US and 2 Israeli pediatric rheumatologists) to independently apply the inclusion criteria and establish the diagnosis of ERA or MRI proved sacroiliitis. Records from a total of 64 subjects from the Israeli medical centers and 72 subjects from the US centers were extracted based on the ICD code given in the pediatric rheumatologic clinic (Supplementary Table 1). Following the process, 39 Israeli and 48 US children and adolescents (overall *N* = 87) met the inclusion criteria (Fig. 1). Eligibility criteria were verified by the coordinating center (Schneider Children’s Medical Center).

### Clinical characteristics

The baseline visit was defined as the first rheumatology appointment. Each of the elements that was documented is routinely recorded in the medical chart in all the four centers once ERA diagnosis is considered. All follow up visits which necessitated a change in treatment were also documented. The data that were collected included the following: demographics (gender, race and ethnicity, symptom onset age and age at diagnosis); family history of autoimmune disease in a first degree relative; clinical signs, symptoms, and physical examination findings (including inflammatory back pain, enthesitis, peripheral arthritis, sacroiliac joint tenderness, limited lumbosacral mobility as represented by modified Schober tests, FABER test, extra articular manifestations (inflammatory bowel disease (IBD), dactylitis, uveitis, and AAU)); laboratory findings (HLA-B27 positivity, anti-nuclear antibody (ANA), C-reactive protein (CRP), erythrocyte sedimentation rate (ESR); radiologic findings (magnetic resonance imaging (MRI) evidence of active or damaged SI joints), and systemic medications prescribed on presentation and upon disease course (including Nonsteroidal Anti-inflammatory Drugs (NSAIDs), Disease-modifying antirheumatic drugs (DMARDs), and biologic therapy with TNF blockade).

Peripheral arthritis was defined as joint pain, swelling or limited range of motion plus tenderness. Enthesitis was defined as tenderness to palpation on entheses insertions. A positive modified Schober test was defined as an increase of less than 6 cm between two lines: 5 cm below and 10 cm above the line joining the dimples of Venus (as a landmark for the lumbosacral junction), when the patient is fully flexed attempting to touch his toes compared to standing position. An increased CRP level was defined as greater than 5 mg/L, and an increased ESR level was defined as greater than 20 mg/h. Positive MRI findings of active SI joints were defined as bone marrow edema and joint space enhancement, and positive MRI findings of chronic sacroiliitis were defined as erosions and/or sclerosis.

As an additional quality assurance, an Inter-Center Comparison of MRI interpretations was performed by comparing the interpretations of trained musculoskeletal (MSK) radiologists for a random sample of 20 MRIs, 10 from each country (US and Israel). The 10 randomly selected MRIs from one country were interpreted blinded to the initial interpretation by a radiologist from the other country (JM, YL), aiming to calculate the level of agreement between the musculoskeletal radiologists in respect to the presence or absence of active and chronic findings. Inter-center comparison of MRI interpretations yielded a matching of 95% (kappa = 0.9). In more details, 11 MRIs were interpreted as positive by the US and Israeli MSK radiologists, 8 MRIs were interpreted as negative by both MSK radiologists and one MRI was interpreted as positive by the American radiologist as compared to the Israeli radiologist, who interpreted in as negative.

In addition to the comparison of the clinical and demographic features between the Israeli and the US cohorts, we examined how many patients from each cohort fulfilled each condition, i.e., ERA or JAS. For that purpose, patients from each cohort were grouped according to one or more of the following phenotypes: (1) ERA as defined by ILAR criteria, (2) JAS defined as sacroiliitis visualized on MRI and symptom onset age < 16 years as accepted [14, 15]. Additional comparison of HLA-B27 positivity and male to female ratio was performed based on SI joint involvement, regardless of the country of origin. In addition, we performed a sub analysis including only the patients with axial involvement upon presentation from each cohort, to compare the age at symptom onset and the age at diagnosis.

### Analysis

The data were analyzed using BMDP software [16]. Fisher’s exact test (two-tailed) was used as appropriate for analysis of between-group differences in discrete variables, and Analysis of Variance (ANOVA) was used for comparison of continuous variables. Analysis of Covariance (ANCOVA) was used to make age adjustment between the two groups. A *P* value of ≤0.05 was considered significant.

## Results

### Demographic features

Demographic features of Israeli vs. US patients are depicted in Table 1 (*N* = 87). Patients were predominantly male of Caucasian ethnicity. Although there was about 20% difference in males’ fraction between the cohorts (56.4% vs. 75% in the Israeli vs. US cohort), the difference was not statistically significant (*p* = 0.11). As compared to the US patients, Israeli patients were significantly older upon presentation (12.3 ± 2.6 and 10.9 ± 2.5 years old in the Israeli and the US cohorts, respectively, *p* = 0.01) as well as at diagnosis (14.3 ± 2.7 and 11.9 ± 2.5 years old in the Israeli and the US cohorts, respectively, *p* < 0.001). A two-fold increase in prevalence of family history of HLA-B27-associated diseases was not statistically significant (5.1% in the Israeli cohort and 10.4% in the US cohort, *p* = 0.45).

**Table 1:** Demographic and clinical features of patients from both the Israeli and the US cohorts upon presentation

Characteristic	All subjects (***n*** = 87)	Israeli cohort (***n*** = 39)	US cohort (***n*** = 48)	*P* value*
**Demographics, mean (SD) or n (%)**				
Sex, male	58 (66.7)	22 (56.4)	36 (75)	0.11
Race, Caucasian	58/78 (74.3)	29/31 (93.5)	29/47 (61.7)	**0.001**
Age at symptom onset in years	11.6 (2.6)	12.3 (2.6)	10.9 (2.5)	**0.01**
Age at diagnosis in years	13 (2.8)	14.3 (2.7)	11.9 (2.5)	**< 0.001**
FHx of HLA-B27-associated disease (FDR)	7 (8)	2 (5.1)	5 (10.4)	0.45
**Clinical characteristics, n (%)**				
Back pain	40 (46)	23 (59)	17 (35.4)	**0.033**
Sacroiliac tenderness	27 (31)	19 (48.7)	8 (16.7)	**0.002**
Positive modified Schober test	13/65 (20)	7/17 (41.2)	6 (21.5)	**0.029**
Positive FABER test	12/66 (18.2)	6/18 (33.3)	6 (12.5)	0.07
Enthesitis	22 (25.3)	3 (7.7)	19 (39.6)	**0.001**
Peripheral arthritis	61 (70.1)	17 (43.6)	44 (91.7)	**< 0.001**
Arthritis in a male over 6 years old	58 (66.7)	22 (56.4)	36 (75)	0.11
Acute symptomatic uveitis	3 (3.4)	0 (0)	3 (6.2)	0.25
HLA-B27 positivity	40/70 (54.8)	8/25 (32)	32 (66.7)	**0.007**
ANA- positive	19/73 (26)	10/28 (35.7)	9/45 (20)	0.17
CRP, elevated	36/74 (48.6)	17/30 (56.7)	19/44 (43.2)	0.34
ESR, elevated	30/62 (48.4)	9/17 (52.9)	21/45 (46.7)	0.78
MRI defined Sacroiliitis	40/67 (59.7)	29/30 (96.7)	11/37 (29.7)	**< 0.001**
**Treatment before or at time of diagnosis, n (%)**				
NSAIDs	72 (82.8)	27 (69.2)	45 (93.7)	**0.004**
DMARDs (sulfasalazine or methotrexate)	12 (13.8)	8 (20.5)	4 (8.3)	**0.13**
Biologics	7 (8)	3 (7.7)	4 (8.3)	1

*P-value of < 0.05 is considered statistically significant. *SD* Standard deviation, *IL* Israel, *FHx* family history, *HLA* Human leukocyte antigen, *FDR* first degree relative, *FABER* Flexion Abduction External Rotation, *ANA* Antinuclear antibody, *CRP* C-reactive protein, *ESR* Erythrocyte sedimentation rate, *MRI* Magnetic resonance imaging

Once restricting the analysis to patients with axial involvement upon presentation, supported by MRI findings (26 Israeli and 6 US patients), the mean age for symptom onset and for time of diagnosis were not statistically significant (12.4 ± 2.5 vs. 11.8 ± 2.9 and 14.5 ± 2.9 vs. 13.3 ± 2.3 for the Israeli and US cohorts, respectively).

### Clinical features

Axial symptoms and signs including inflammatory back pain, SIJ tenderness, and positive modified Schober test were significantly more prevalent among Israeli patients upon presentation as compared to the US patients (59% vs. 35.4, 48.7% vs. 16.7, and 41.2% vs. 21.5%, respectively, all *p* < 0.05), whereas peripheral arthritis and enthesitis were significantly more prevalent among the US patients (43.6% vs. 91.7 and 7.7% vs. 39.6%. respectively, *p* ≤ 0.001 (Table 1)). Positive FABER tended to be more common in the Israeli cohort as compared to the US cohort but did not reach significance (33.3% vs. 12.5%, *p* = 0.07). Presence of the HLA-B27 was more frequently seen in US than Israeli patients (66.7% vs 32% *p* = 0.007). The presence of Sacroiliitis defined by MRI was significantly higher in the Israeli cohort (96.7%, (29/30)) as compared to the US cohort (29.7%, (11/37); *p* < 0.001), with the presence of pelvis MRI from time of presentation being 77% for both cohorts.

### Criteria fulfilled upon presentation

**Fig. 2:**
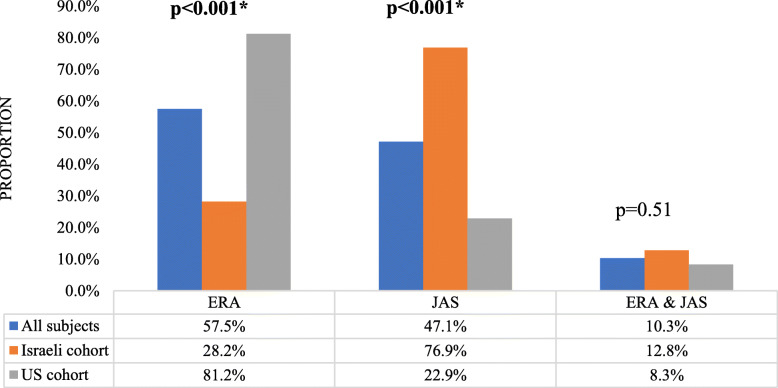
Criteria fulfilled upon disease presentation by country. *ERA* Enthesitis-related arthritis, *JAS* Juvenile ankylosing spondylitis. * Statistically significant differences (*p* < 0.05) between groups

Patients were classified as ERA by ILAR criteria, JAS, or both (Fig. 2). US patients frequently met criteria for ERA at presentation as opposed to Israeli patients (39 (81.2%) versus 11 (28.2%), *p* < 0.001). In contrast, Israeli patients usually met criteria for JAS (30 (76.9%) vs. 11 (22.9%), p < 0.001). Fulfillment of both ERA and JAS criteria was similar between Israeli and US cohorts (12.8% vs. 8.3%, *p* = 0.51).

### Characteristic differences by axial involvement

Overall 77% of each cohort (30 from Israel and 37 from the US, overall *N* = 67) had MRI imaging upon presentation, 57 of which had a known HLA-B27 status. Table 2 demonstrates the gender differences and the HLA-B27 status based on sacroiliac involvement upon presentation to pediatric rheumatology. More than half of the patients in both groups were males, however this proportion was much higher among those without axial involvement (77.8%) as compared to those with axial involvement (55%, *p* = 0.07). Upon presentation, a positive HLA-B27 status was significantly more prevalent among patients without sacroiliac involvement (74.1%) as compared to merely 30% of patients who presented with sacroiliitis (*p* = 0.001).

**Table 2:** Characteristics by axial involvement on MRI

Characteristic, n (%)	All subjects (*n* = 67)	Positive sacroiliitis on MRI	Negative sacroiliitis on MRI	*P* value
Gender, male	43 (64.2)	22 (55)	21 (77.8)	0.07
HLA-B27 positivity (*n* = 57)	29 (50.9)	9 (30)	20 (74.4)	**0.001**

Gender distribution and HLA-B27 positivity by axial involvement upon presentation. *MRI* Magnetic resonance imaging, *HLA* Human leukocyte antigen

### Treatment

Most patients were taking NSAIDs)Ibuprofen, Naproxen, Indomethacin) upon presentation to pediatric rheumatology or were prescribed NSAIDs at time of diagnosis by pediatric rheumatology, with a significantly greater proportion among the US compared to the Israeli patients (93.7% vs. 69.2%, *p* = 0.004). An additional 20.5% of Israeli vs. 8.3% of US patients were prescribed DMARDs (Methotrexate, Sulfasalazine) at the time of diagnosis and 7.7% vs. 8.3% were prescribed biologic (TNF inhibitors) agents at that time (Table 1).

## Discussion

In this study we find that Israeli JSpA patients demonstrate different features compared to the characteristics described in other populations worldwide. Most Israeli patients presented to the rheumatology clinic with axial symptoms rather than peripheral symptoms (including enthesitis). Peripheral symptoms of arthritis and enthesitis were the dominant presentation of the US group and were comparable with other reports [10–12, 17–21]. These observations are supported by the significantly higher percentage of sacroiliitis on MRI upon presentation in the Israeli versus the US cohort (96.7% vs 29.7%), which were validated by the inter-center comparison, with an excellent level of agreement between the reading radiologists in the two countries. Weiss et al. [19] showed in a multi-center international inception cohort of children with ERA, including four American centers and one Italian site, that more than 90% of ERA subjects presented with peripheral arthritis at the time of diagnosis, similarly to the US cohort in our study (91.7%). In addition, enthesitis was also a dominant presenting symptom, with more than 70% at time of diagnosis, which was also similar to our results, though our US cohort presented with lower percentage of enthesitis (39.6%) [19]. Additional cohorts of children with ERA from France [17] and India [18] revealed that most patients present with peripheral, rather than axial, symptoms. A recent Japanese study which compared Japanese, Asian and non-Asians adult patients with SpA demonstrated differences in the distribution of peripheral SpA, axial SpA and HLA B27 among these populations, which may indicate that the presentation of the disease is essentially affected by ethnic and environmental factors [20]. In light of the above, the lack of peripheral arthritis and enthesitis in most of our Israeli cohort may be attributed to genetic variations among the different ethnic populations in Israel [22]. In addition, not only genetic factors but also environmental ones such as the role of microbiome, may play a role in the difference between the Israeli and US population [22, 23].

The ages at symptom onset and at diagnosis in the Israeli cohort were significantly higher compared to US cohort. The mean age at diagnosis of the Israeli cohort, 14.3 ± 2.7 years, was also higher than reported for ERA and JAS cohorts worldwide, in which the mean age at diagnosis has been reported to be 10 to 13 years [24–28]. In respect to the time from symptom onset to diagnosis, there was also a significant delay of 2 years in the Israeli cohort as compared to 1 year in the US cohort (*p* = 0.003). This delayed presentation of Israeli children to the rheumatologic clinic may be partially explained by our results that show a much higher percentage of enthesitis in the US cohort, which is known to be a painful symptom which usually urge patients to seek medical care. It may also be related to differences in referral patterns. Interestingly, once restricting the analysis to include only patients with axial involvement upon presentation, no significant difference was found between the age at symptom onset and diagnosis between the two cohorts. This may explain the overall differences in age of symptom onset and age of diagnosis, as the Israeli cohort is comprised from a significant percentage of patients with axial disease as compared to the US cohort.

Although a major etiologic factor in ERA and JAS is believed to be related to HLA-B27 [22, 23], in our study, the prevalence of HLAB27 in the JSpA Israeli cohort was only 50% of the prevalence in the US cohort. This may indicate a different genetic profile of the Israeli population that modulates the disease category. Interestingly, a study which aimed to investigate the distribution of HLA classes I and II both serologically and by oligo-typing in a group of Israeli patients with psoriatic arthritis (PsA) revealed that, as in rheumatoid arthritis, Israeli patients with PsA present a different HLA distribution as compared to worldwide populations, including the US population [29]. Indeed, in our study, a comparison of HLA-B27 status based on the MRI evidence of sacroiliitis, a proxy for axial involvement, revealed that the HLA-B27 positivity was significantly more prevalent among patients who presented with peripheral disease (74.4% vs. 30%, *P* = 0.001). Although the overall percentage of HLAB27 in the US population is 6.1% [30], the Israeli percentage of HLAB27 is unknown.

Interestingly, the use of NSAIDs upon presentation to pediatric rheumatology and time of diagnosis was significantly lower in the Israeli cohort (69.2% vs 93.7%, *p* = 0.004). These differences may be explained by delayed administration of NSAIDs among Israeli patients due to a different disease phenotype, who, in many cases, present to the clinic with indolent axial symptoms, such as inflammatory back pain and without an available MRI test, which is required to establish the diagnosis, and therefore, are not given any medication until the diagnosis is supported by MRI. The fact that the US cohort presented with more peripheral arthritis and enthesitis, a diagnosis that can be established clinically by a musculoskeletal exam, might explain the higher usage of NSAIDs upon the time of diagnosis. Another possible explanation to the lower percentage of NSAIDs administration at time of diagnosis in the Israeli cohort is that since more than 20% of Israeli patients were prescribed with a DMARD (in case of coexisting peripheral disease) and/ or biologic agents (in cases of definite axial disease), NSAIDs were avoided.

Our study reveals that most Israeli patients diagnosed with JSpA demonstrate different features compared to a US population. Most of this Israeli cohort do not meet the ILAR criteria for diagnosis of ERA, but mainly manifest with axial presentation– i.e. sacroiliitis. Consequently, applying the ERA ILAR criteria by pediatric rheumatologists in Israel may lead to misdiagnosis or delay in the diagnosis of children with axial involvement. It is important to note that adjusting our results to the difference in age between the two cohorts using ANCOVA did not change the significance of our results.

Our findings should be interpreted in light of several limitations. First, since this was a retrospective study and not an inception cohort, the data collection was not uniform at time of presentation, leading to missing data in multiple features such as HLA-B27 (for which the status of positivity was unknown for some patients), ANA positivity and inflammatory markers. This also made classification more difficult. Second, the study population range period varied among the cohorts due to lack of sufficient number of patients in the Israeli cohort. However, the ERA criteria were published in 2001 and has not been updated since ever, and both cohort of patients were diagnosed after December of 2004 [9]. Third, referral patterns, clinical examination and treatment practices may vary among physicians and sites. However, all Israeli pediatric rheumatologists involved in this study were trained at fellowship programs in North America and have seen and treated North American JSpA patients. Finally, sacroiliac imaging was performed at the discretion of the pediatric rheumatologist based on clinical symptoms consistent with axial involvement. Several reports have shown that sacroiliitis can be frequently present in children who do not complain of back pain [13, 31, 32]. Therefore, the prevalence of MRI-confirmed sacroiliitis in both of our cohorts might underestimate the actual numbers. However, 77% of each of our cohorts did have MRI imaging of the pelvis. It is important to emphasize that this study was not designed to investigate the differences among the cohorts throughout the disease course but only upon presentation.

## Conclusion

In conclusion, this study reveals that the clinical and demographic features of children diagnosed with JSpA in Israel are different compared to a US cohort from a large US center. Israeli children are older upon presentation and time of diagnosis as compared to their US counterparts, are more likely to be HLA-B27 negative and usually present with axial symptoms rather than peripheral arthritis or enthesitis, which are both significantly more common in the US cohort. Further studies are needed to explore the genetic and environmental factors that are associated with our findings.

## Supplementary Information

**Additional file 1: Supplementary Table 1.** ICD 9 and ICD 10 codes used for medical records.

## Data Availability

The datasets analyzed during the current study available from the corresponding author on reasonable request.

## Abbreviations

JSpA: Juvenile Spondyloarthritis
ERA: Enthesitis Related Arthritis
JAS: Juvenile Sacroiliitis
JIA: Juvenile Idiopathic Arthritis,
HLA B27: Human Leukocyte Antigen 27
US: United States
ILAR: International League against Rheumatism
