# Supplementary material for: Do geography and ethnicity play a role in juvenile Spondyloarthritis? A multi-center binational retrospective study
Source: Pediatr Rheumatol Online J. 2021 Jan 6;19:4. doi: 10.1186/s12969-020-00489-8 (PMC7788991; doi:10.1186/s12969-020-00489-8)
Supplement: Supplementary file 1 — Additional file 1: Supplementary Table 1. ICD 9 and ICD 10 codes used for medical records. [file 12969_2020_489_MOESM1_ESM.docx]

| **ICD 9 Codes** | ICD 714.3 (JIA) |
| --- | --- |
|  | ICD 720.2 (Sacroiliitis) |
|  | ICD 720.9 (Spondyloarthropathy) |
|  | ICD 720.0 (Ankylosing Spondylitis) |
| **ICD 10 Codes** | ICD M08.8 (Enthesitis Related Arthritis, JIA or undifferentiated arthritis) |
|  | ICD M46.1 (sacroiliitis) |
|  | ICD M45.9 (ankylosing spondylitis) |

**Supplementary table 1:** ICD 9 and ICD 10 codes used for medical records identification**.**
